# Photobiomodulation augments the effects of mitochondrial transplantation in the treatment of spinal cord injury in rats by facilitating mitochondrial transfer to neurons via Connexin 36

**DOI:** 10.1002/btm2.10473

**Published:** 2022-12-21

**Authors:** Zhijie Zhu, Xin Li, Xuankang Wang, Xiaoshuang Zuo, Yangguang Ma, Xue Gao, Zhuowen Liang, Zhihao Zhang, Zhiwen Song, Tan Ding, Cheng Ju, Penghui Li, Kun Li, Jiawei Zhang, Huilin Quan, Zhe Wang, Xueyu Hu

**Affiliations:** ^1^ Department of Orthopedics Xijing Hospital, Fourth Military Medical University Shaanxi China; ^2^ 967 Hospital of People's Liberation Army Joint Logistic Support Force Dalian Liaoning China

**Keywords:** connexin, mitochondrial transplantation, neuron, photobiomodulation, spinal cord injury

## Abstract

Mitochondrial transplantation is a promising treatment for spinal cord injury (SCI), but it has the disadvantage of low efficiency of mitochondrial transfer to targeted cells. Here, we demonstrated that Photobiomodulation (PBM) could promote the transfer process, thus augmenting the therapeutic effect of mitochondrial transplantation. In vivo experiments, motor function recovery, tissue repair, and neuronal apoptosis were evaluated in different treatment groups. Under the premise of mitochondrial transplantation, the expression of Connex36 (Cx36), the trend of mitochondria transferred to neurons, and its downstream effects, such as ATP production and antioxidant capacity, were evaluated after PBM intervention. In in vitro experiments, dorsal root ganglia (DRG) were cotreated with PBM and 18β‐GA (a Cx36 inhibitor). In vivo experiments showed that PBM combined with mitochondrial transplantation could increase ATP production and reduce oxidative stress and neuronal apoptosis levels, thereby promoting tissue repair and motor function recovery. In vitro experiments further verified that Cx36 mediated the transfer of mitochondria into neurons. PBM could facilitate this progress via Cx36 both in vivo and in vitro. The present study reports a potential method of using PBM to facilitate the transfer of mitochondria to neurons for the treatment of SCI.

## 1 INTRODUCTION

Spinal cord injury (SCI), as a kind of disability disease with a high incidence worldwide, often leads to the impairment of motor and sensory functions in patients. At present, its treatment is still a thorny problem. As the “powerhouse” of cells, mitochondria participate in various fundamental biological processes and its damage has been considered an important cause of neuronal injury in SCI.^1^, ^2^, ^3^ Neurons primarily rely on mitochondrial oxidative phosphorylation (OXPHOS) to meet their high energy requirements.^4^ Adenosine triphosphate (ATP) synthesis in mitochondria is crucial for membrane potential maintenance, nerve conduction, and synaptic plasticity.^5^ In addition, axonal injury triggers acute stress, which reduces mitochondrial transportation to the growth cones of neurons for sprouting, thereby causing axonal energy deficiency and axonal regeneration failure.^6^, ^7^

There is mounting evidence that directly supplementing mitochondria in the damaged area, which is called mitochondrial transplantation, has become an attractive treatment strategy. Compared with the SCI group, the mitochondrial treatment group partially improved motor function recovery, and histological repair and reduced the inflammatory response and neuronal apoptosis.^8^, ^9^, ^10^ It is noteworthy that this strategy still encounters two main problems. First, what is the most suitable source of exogenous mitochondria for transplantation? Autologous liver, heart, and muscle tissues are rich in mitochondria. However, the extraction of these mitochondria results in tissue injury and can only be performed once, limiting the application of this approach.^11^ Cell lines such as stem cells and PC‐12 cells are the common sources of transplantation, but ethical issues should be carefully considered before clinical practice.^12^ Second, how to efficiently facilitate exogenous mitochondria transfer, particularly to neurons. Even in the case of mitochondrial transplantation, the proportion of exogenous mitochondria entering neurons was still not high, let alone the proportion of endogenous astrocyte mitochondria transferred to neurons in the absence of exogenous mitochondria.^11^, ^13^ Moreover, macrophages, microglia, and astrocytes can also ingest mitochondria but whether they play a beneficial role after SCI is still unclear.^9^, ^14^, ^15^, ^16^, ^17^

For the first problem, platelet‐derived mitochondria are the suitable candidates. The content of platelets in peripheral blood is high, mitochondria could be isolated many times, and the trauma caused by simple venipuncture is less than that caused by muscle mitochondria separation. More importantly, there are no issues related to ethics or immune exclusion, so this treatment strategy has broad prospects for clinical application.^11^ For the second problem, we focused on the particularity mechanism of mitochondrial transfer to neuron‐gap junctional intercellular communication (GJIC). It is formed by the connexin of a donor cell and a recipient cell that allows ions, signaling molecules, and metabolic molecules to move in and out of cells.^18^ The connexin from a single cell can also form a hemichannel and mediate the exchange of materials between the cell and the extracellular environment.^19^ In an in vitro study, inhibition of Connexin 43 could reduce mitochondrial metastasis to VSC4.1 motor neurons, while activation of Connexin 43 increases mitochondrial metastasis.^9^ However, VSC4.1 motor neurons are different from primary neurons, so further research is needed to demonstrate the specific pathway by which mitochondria enter neurons. Connexin 36 (Cx36) is a specific connexin that only expresses on the surface of neurons and primary neuron–dorsal root ganglia (DRG) but not on other cells, such as astrocytes and macrophages.^20^, ^21^, ^22^ Therefore, we speculated that Cx36 could be the key point of mitochondrial transfer to neurons and DRGs which might provide a novel insight into the treatment of SCI.

Studies have shown that prolonged dark adaptation decreased the expression of Cx36 in the retina while increasing laser exposure promoted Cx36 expression.^23^, ^24^ Photobiomodulation (PBM), also called lower‐level laser therapy, has attracted much attention in the past 10 years. Its main principle is that cytochrome c oxidase absorbs photons of near‐infrared laser to promote mitochondrial bioenergetic and final ATP production.^25^ In addition, PBM could regulate reactive oxygen species (ROS) production and activate a variety of transcription factors thus it has been used to treat SCI, ischemic stroke, traumatic brain injury, Alzheimer's disease and so on.^26^, ^27^, ^28^, ^29^ However, mitochondrial dysfunction that occurs after SCI limits the therapeutic effect of PBM.^30^

To address the problems mentioned above, we first studied embedded laser fiber‐mediated PBM combined with platelet‐derived mitochondrial transplantation in the treatment of SCI. Our results indicated that combined treatment was better than that of a single treatment in terms of motor function recovery, tissue repair, and inhibition of neuronal apoptosis. Next, we found PBM enhanced the expression of Cx36 in the spinal cord and promoted the transfer of mitochondria into neurons, as well as the specific downstream effects such as ATP production and antioxidant capacity. Finally, we verified our speculation by using an inhibitor of Cx36 on DRGs in vitro.

## 2 MATERIALS AND METHODS

### 2.1 Platelet‐derived mitochondrial isolation and analysis by flow cytometry

The protocol for platelet and mitochondrial isolation was performed as previously described.^31^ Ten milliliters of rat blood were centrifuged twice at room temperature for 5 min each to take out cell debris. The supernatant was centrifuged at 800×*g* for 10 min to collect a white pellet, that is, platelet granules. Then, 5 ml of prechilled GENMED lysis working solution (GMS10062.2, GenMed Scientifics, Inc., USA) was added, and the slurry was homogenized. The samples were centrifuged at 4°C for 10 min at a speed of 1500×*g* to remove the remained cells. The supernatant was aspirated and centrifuged at 4°C for 10 min at a speed of 11,000×*g* to obtain mitochondria. A mitochondrial storage solution (C3609, Beyotime, China) was added to resuspend the mitochondria. Platelet purity as determined using flow cytometry (Beckman F500, USA): APC anti‐mouse/rat CD42d antibody (148,505, BIOLEGEND, USA) was added. Samples without antibodies were used as negative controls, and parameters such as the gating strategy and voltage were set using the negative controls.^32^ Mitochondria count: mitochondria were pre‐stained with Mito Tracker red CMXRos (M7512, Invitrogen, USA) and then mixed with green fluorescent microspheres. Unstained mitochondria and microspheres only were used as negative controls to set the gating strategy and voltage.^31^ The specific results are shown in Supplementary Figure 1.

### 2.2 JC‐1 assay

The mitochondrial membrane potential was measured by the Enhanced Mitochondrial Membrane Potential Detection Kit (C2003S, Beyotime, China). Carbonyl cyanide 3‐chlorophenylhydrazone (CCCP) is an uncoupler of mitochondrial oxidative phosphorylation that led to the loss of the mitochondrial membrane potential. The control group was treated with 50 μM CCCP (C6700, Solarbio, China) for 20 min, and JC‐1 fluorescence was detected.

### 2.3 Spinal cord injury model

All animal experiments are approved by the Animal Ethics Committee of the Fourth Military Medical University (IACUC‐20220401). The rats were randomly divided into six groups: the sham group, SCI group, SCI+Vehicle group, SCI+Mito group, SCI+PBM group, and SCI+Mito+PBM group. A modified bilateral spinal cord clamping technique was used to model SCI as previously described.^33^ The lamina of T10 was removed to expose the spinal cord. SCI was induced by clamping the T10 spinal cord with forceps for 40 s. The sham group only opened the lamina without clamping the spinal cord. The criteria for successful modeling: rats performed rapid retraction like shaking of the whole body, rapid edema and congestion of the local spinal cord surface, and the dura mater remaining intact.

### 2.4 Mitochondrial injection

The SCI+Mito group was injected with 10 μl of mitochondria in mitochondrial storage solution using a micro syringe and stereotaxic device as in the previous study.^9^ According to the research of others,^9^, ^34^ we conducted a pre‐experiment and determined that 3 × 10^5^ mitochondria per individual are the best dose. The Mito group in the main body of this article specifically refers to 3 × 10^5^ mitochondria per individual group. The SCI group was injected with 10 μl of mitochondrial storage solution.

### 2.5 Photobiomodulation therapy

Laser fibers were embedded in the SCI+Vehicle group, SCI+PBM group, and SCI+Mito+PBM group as in the previous study^33^: the front and rear end of the fiber is stitched sequentially on the T8 and T12 spinous protrusions to ensure that the laser could reach T10 spinal cord to carry out PBM. Studies have shown that a laser with a wavelength between 700 and 770 nm has a weak stimulating effect on organisms. A laser with a wavelength >1200 nm is significantly absorbed by water molecules, and a laser with a wavelength of approximately 810 nm has the best therapeutic effect.^35^, ^36^ Meta‐analysis results suggest that 14 days of PBM could better restore motor function after SCI.^37^ Therefore, we choose an 810 nm laser to carry out PBM for 14 days. PBM was performed once a day for 60 min. Rats were irradiated using an 810 nm semiconductor laser (MW‐GX‐808, China Lei Shi Optoelectronics Co., Ltd., 810 nm wavelength, 150 mW output power). More details about the animal model are shown in Supplementary Figure 2 and Supplementary Table 1.

### 2.6 Gait analysis

For gait analysis, the soles of the hindlimbs were dyed with blue ink, and the dorsal part of the hindlimb was dyed with red ink. Then, the animals were allowed to freely walk down a runway (20 cm wide and 50 cm long) covered with white paper, and researchers calculated the stride length and stride width according to previous methods and definitions.^9^, ^38^

### 2.7 Tissue preparation

Rats were infused with 4% paraformaldehyde (PFA) through the heart, and an approximately 2 cm long piece centered on the site of the injury was obtained. The tissue was embedded in an optimal cutting temperature compound, cut into continuous sagittal or horizontal slices at a thickness of 7 μm, and stored at −20 °C.

### 2.8 Nissl staining

Tissue sections were treated with toluidine blue staining solution (G1032, Servicebio, China) for 5 min. Then, they were treated with 1% glacial acetic acid, incubated in xylene for 10 min, and sealed with neutral gum. The number of neuronal cell bodies at high magnification was determined by Image J software.

### 2.9 Luxol fast blue (LFB) staining

Tissue slices were placed in myelin staining solution A (G1030, Servicebio, China) for 1 h, immersed in myelin dye B for slight differentiation for 2 s, immersed in myelin dye C for 15 s, and washed until the myelin sheaths were blue and the other components were almost colorless. Then, the sections were dehydrated with absolute ethanol and sealed. The percentage of blue area at high magnification was calculated with Image J software.

### 2.10 Immunofluorescence

The slices were treated with 0.3% Triton X‐100 for 20 min and were blocked with serum for 1 h. Then the slices were incubated with primary antibodies overnight at 4 °C. The following primary antibodies were used: TOM20 (1:400, 11802‐1‐AP, Proteintech, China), anti‐MAP2 (1:400, 8707T, Cell Signaling Technology, USA), anti‐NeuN (1:400, ab104224, Abcam, UK), anti‐beta III Tubulin (1:200, ab78078, Abcam, UK), polyclonal anti‐Cx36 (1:200, 51‐6200, Thermo Fisher, USA), MAP2 Monoclonal Antibody (1:200, 67015‐1‐Ig, Proteintech, China), Anti‐HO‐1 (1:400, ab189491, Abcam, UK). The sections were incubated with a secondary antibody (1:400) at room temperature for 1 h and finally stained with DAPI. Images were obtained using a fluorescence microscope (BX53, Olympus Corporation, Japan) and a confocal microscope (Nikon ECLIPSE Ti‐S, Japan). Panoramic Viewer software (3DHISTECH, Hungary) was used to obtain images of the full spinal cord. The MAP2 fluorescence intensity at high magnification was calculated by Image J.

### 2.11 TUNEL staining

TUNEL staining solution was prepared using a TUNEL kit (C1090, Beyotime, China). The slices were incubated with a secondary antibody and TUNEL staining solution for 1 h. The ratio of TUNEL positive neurons was calculated by Image J.

### 2.12 Image analysis and quantification

For each rat, three discrete slices were selected near the central canal of the spinal cord with intervals of 100 μm. Pictures were collected in the bilateral areas 200 μm rostral and caudal to the lesion site. Based on the previous analysis method of our group, empirical methods are used for calibration to ensure that unbiased data are collected.^33^, ^39^, ^40^ Cell counts in each section were averaged from five independent fields randomly selected within the symmetric cephalad and caudal sides of the lesion center for analysis. All analytical quantifications were performed by independent experimenters.

### 2.13 Western blotting

The rats were perfused with normal saline, and a 1 cm piece of spinal cord tissue centered on the site of the injury was collected, homogenized, and lysed in the RIPA lysis solution. The supernatant was collected, the bicinchoninic acid (BCA) method was used to determine the protein concentration, and the proteins were boiled, loaded, separated by electrophoresis, and transferred onto a membrane. The membrane was blocked in skimmed milk powder and incubated with the appropriate primary antibody (see Supplemental Table 2) at 4 °C overnight. The membrane was incubated with a secondary antibody (1:2000) at room temperature for 1 h, and developed by enhanced chemiluminescence.

### 2.14 Transmission electron microscopy (TEM)

After the tissue was fixed by glutaraldehyde and 1% osmium tetroxide, alcohol was dehydrated. Embedded with EPON812 resin, made into slices, and then stained for observation. The sections were examined with a HITACHI transmission electron microscope. G‐ratio refers to the ratio of the inner diameter to the outer diameter of each axon fiber in about the same 150 axons of five rats in each group and it was calculated by Image J.^41^

### 2.15 Analysis of oxidative stress and ATP levels

The tissue was homogenized and centrifuged, and the supernatant was aspirated. The MDA, 3‐NT, and 8‐OHdG contents in the supernatant were analyzed using an enzyme‐linked immunosorbent assay (ELISA) kit (Meimian Industrial, China). The ROS content in the supernatant was analyzed using a ROS kit (BB‐470512, BestBio, China). The ATP content in the supernatant was analyzed by an ATP analysis kit (S0027, Beyotime, China).

### 2.16 Primary neuronal culture

DRG was obtained from SD rat neonates (P0–P2) according to the preliminary research of our group.^42^ Briefly, microforceps were used to separate bilateral DRGs under a microscope. Then it was shredded, and trypsin (0.25%, Beyotime, China) and collagenase IV (0.2%, Sigma, USA) were added for digestion for 1 h. DRG neurons were resuspended in a neurobasal medium (Gibico, USA) containing B27, Glutamine, and penicillin/streptomycin. The medium was changed at 24 h and then carried out the next step.

### 2.17 Oxygen and glucose deprivation (OGD) and processing

DRG injuries were induced by OGD and reoxygenation to mimic ischemic injury of the spinal cord in vivo.^9^ Briefly, cells in the OGD group were incubated for 4 h in glucose‐free D‐Hank's balanced salt solution (11966025, Thermo Fisher, USA) in a sealed hypoxic GEN bag (BioMèrieux, Marcy I'etoile, France) equipped with a catalyst. After OGD ended, changed back to a normal medium and incubated under normal conditions for 24 h until the next progress. To explore whether connexin mediates the transfer of mitochondria into neurons, we dissolved the connexin inhibitor 18β Glycyrrhetic acid (18β‐GA) in DMSO as previously described.^43^ DRG cells were preincubated with 18‐β‐GA (50 μM, SE8280, Solarbio, China) for 8 h and then cocultured for 24 h with exogenous mitochondria.^9^ To promote connexin function, the cells were exposed to laser twice per day for 10 min at 9 a.m. and 9 p.m. The growth of neurites was measured with Image J software and the plug‐in Neuron J according to the previous methods.^27^

### 2.18 Statistical analysis

All experiments were repeated at least three times independently, and all data are expressed as the mean ± SD. GraphPad Prism 8 software was used for statistical analysis and graphing. For two groups comparison, a t‐test was used. For multiple comparisons, the data were analyzed by one‐way ANOVA followed by Bonferroni's post hoc test. Repeated measurement data were compared by two‐way repeated‐measures ANOVA and Bonferroni's test. *p* < 0.05 was considered statistically significant. *p < 0.05, **p < 0.01, ***p < 0.001. NS is not significant.

## 3 RESULTS

### 3.1 Identification of platelet‐derived mitochondrial properties and optimal dose of transplantation

Flow cytometry analysis indicated that the purity of the platelets was 95% (Supplementary Figure 1a), and approximately 0.93 × 10^6^ mitochondria could be isolated from 10 ml of whole blood (Supplementary Figure 1b). Then, TEM was used to determine the morphological integrity of the isolated mitochondria (Supplementary Figure 1c). Supplementary Figure 1d showed that COX IV (an internal marker of mitochondria) but not β‐tubulin (an internal marker of the cytoplasm) was highly expressed in the purified mitochondrial lysate. Moreover, ATP levels and the membrane potential (red/green ratio) were decreased after CCCP inhibition, which indicated that purified mitochondria were functional (Supplementary Figure 1e).

We then studied the effects of three different doses of mitochondria transplantation on motor function recovery and neuronal apoptosis at 14 dpi. The data showed that compared with the low dose (1 × 10^5^)group, the medium dose (3 × 10^5^) group significantly improved the step length (Supplementary Figure 3a) and reduced cell apoptosis (Supplementary Figure 3b). The therapeutic effect of the high dose group (5 × 10^5^) was not better than that of the medium dose group. Therefore, the medium dose (3 × 10^5^) was selected for subsequent experiments.

### 3.2 Combined therapy promoted motor function recovery and tissue repair at 14 dpi

**FIGURE 1:**
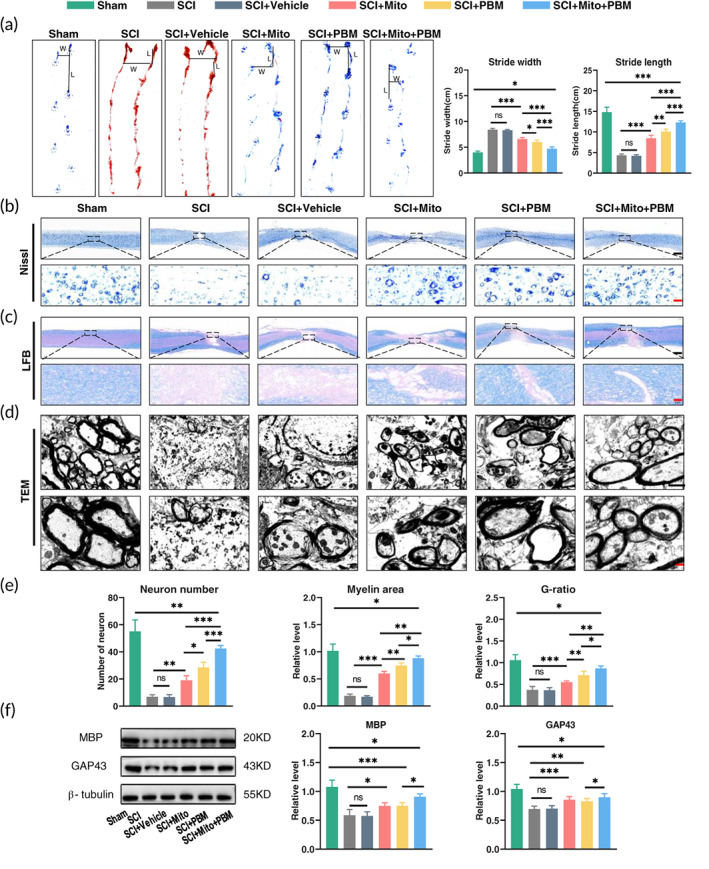
Combined therapy promoted motor function recovery and tissue repair at 14 dpi. (a) Gait analysis and representative gaits of each group. L = stride length, W = stride width. Calculate the average stride width and stride length. *n* = 5. (b) Nissl staining to count the number of motor neurons. Scale bar of upper panel: 1 mm. Lower panel scale bar: 10 μm. *n* = 5. (c) LFB staining was used to observe the remyelination, and the blue area was myelin. Upper panel scale bar: 1 mm, lower panel scale bar: 20 μm. *n* = 5. (d) Axons and myelin were observed by TEM. The lower panel image is a magnification of the upper panel image. Upper panel scale bar: 1 μm. Lower panel scale bar: 500 nm. *n* = 5. (e) Qualitative analysis of the number of motor neurons (b), myelin area (c), and G‐ratio (d) by Image J in each group. *n* = 5. (f) Western blot analysis and quantification of MBP and GAP43 expression levels in each group. *n* = 5.

Gait analysis and tissue repair evaluation were applied to investigate the effect of combination therapy. The stride length was longer and stride width was shorter in the SCI+Mito+PBM group compared with the other two treatment groups (Figure 1a, stride width, *F*(5, 24) = 198.0, *p <* 0.001; stride length, *F*(5, 24) = 205.0, *p <* 0.001). Subsequently, we found that both the number of motor neurons and the myelin area in the SCI group were decreased on day 14 while all treatments increased them, especially in the SCI+Mito+PBM group (Figure 1b, e, number of motors neurons: *F*(5, 24) = 102.2, *p <* 0.001; Figure 1c, e, myelin area: *F*(5, 24) = 168.3, *p <* 0.001). The G‐ratio in the SCI+Mito+PBM group was higher than those in the SCI+PBM group and the SCI+Mito group (Figure 1d, e, *F*(5, 24) = 61.52, *p <* 0.001). The expression of two proteins related to nerve growth, that is, myelin basic protein (MBP) in the SCI+Mito+PBM group was higher than that in the other two treatment groups, while growth‐associated protein‐43 (GAP43) in the SCI+Mito+PBM group were not higher than that in the other treatment groups (Figure 1f, MBP: *F*(5, 24) = 29.66, *p <* 0.001; GAP43: *F*(5, 24) = 30.05, *p <* 0.001).

### 3.3 Combined therapy alleviated mitochondrial‐related neuronal apoptosis at 14 dpi

**FIGURE 2:**
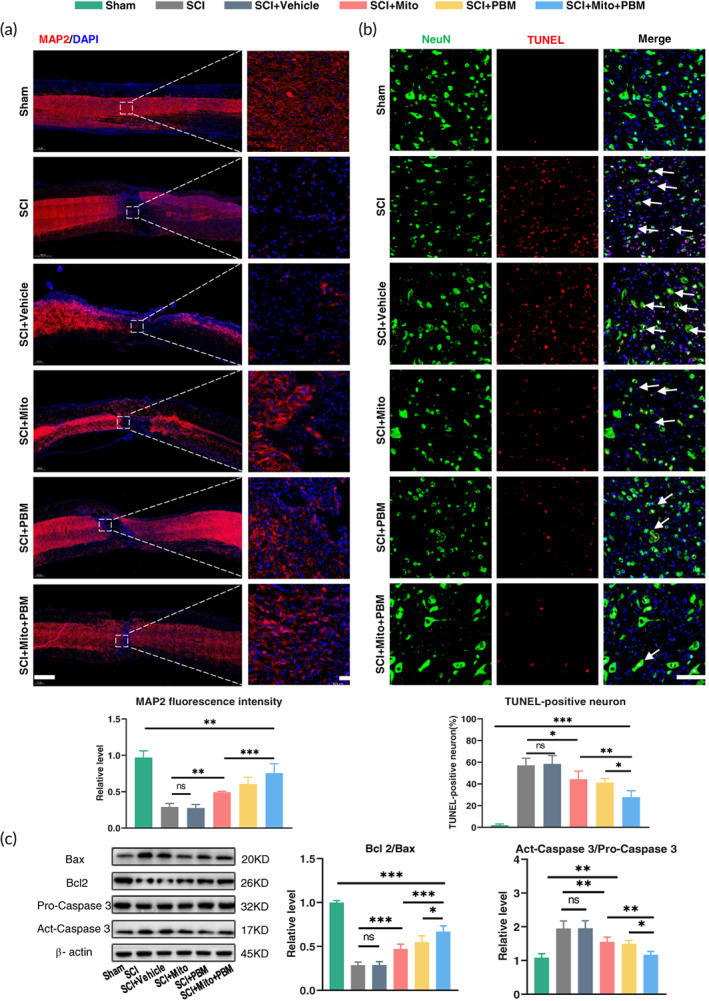
Combined therapy alleviated mitochondrial‐related neuronal apoptosis. (a) Representative images of the whole spinal cord with MAP2 (red) in left panel pictures. Scale bar: 1 mm. The right panel pictures are enlarged from the left one. Scale bar: 50 μm. Calculated the fluorescence intensity of MAP2 in the right panel pictures by Image J. *n* = 5. (b) TUNEL (red) and NeuN (green) co‐stained, nuclei stained with DAPI (blue). Scale bar: 50 μm. The statistical ratio of TUNLE‐positive neurons by Image J, Arrows point to representative cells. *n* = 5. (c) Western blot analysis and quantification of Bax, Bcl2, pro‐caspase 3, and active‐caspase 3 expression levels in each group. *n* = 5.

We noticed that there was a decrease in motor neurons after SCI (Figure 1b, e), so we further investigated whether this decrease was associated with mitochondria damage. At the morphology level, fluorescence staining showed that the level of MAP2 (a maker of neuronal cell bodies and axons) decreased after injury and was reversed by treatment. The expression of MAP2 in the SCI+Mito+PBM group was higher than that in the SCI+Mito group but was not different from that in the SCI+PBM group (Figure 2a). Moreover, treatment with PBM and mitochondria significantly reduced the proportion of apoptotic neurons. The proportion of apoptotic neurons in the SCI+PBM group (41.2 ± 3.7) and SCI+Mito group (44.2 ± 7.62) was significantly lower than that in the SCI group (57.1 ± 2.62) but higher than that in the SCI+Mito+PBM group (27.8 ± 5.99, Figure 2b). We further investigate neuronal apoptosis at the molecular level. When mitochondria damage occurs, an enhancement in the Bax/Bcl 2 ratio triggers the delivery of cytochrome c from mitochondria to the cytoplasm, which activates caspase‐3 and then induces apoptosis.^44^ Our results demonstrated both the Bax/Bcl 2 ratio and the activated caspase‐3/pro Caspase‐3 ratio decreased after treatment especially in the SCI+Mito+PBM group (Figure 2c: Bax/Bcl 2 *F*(5, 24) = 27.56, *p <* 0.001; activated caspase‐3/pro Caspase‐3: *F*(5, 24) = 140.3, *p <* 0.001).

### 3.4 In the context of mitochondrial transplantation, PBM promoted the expression of Cx36, KIF5B, TRAK2, and Rhot1 and reduced the expression of SNPH

The above results proved that the therapeutic effect of combined treatment was better than that of a single treatment, hence we began to explore the potential mechanisms of combination therapy. We explored Cx36, which might mediate mitochondrial transfer into neurons and some proteins related to mitochondrial transportation to axons after mitochondrial transplantation. KIF5B, TRAK1/2, and RhoT1/2 GTPases could cooperate directly to facilitate mitochondrial transportation and then promote axon growth.^45^, ^46^ In addition, the anchoring protein syntaphilin (SNPH) inhibits mitochondrial movement and then hinders axonal regeneration.^7^, ^47^

**FIGURE 3:**
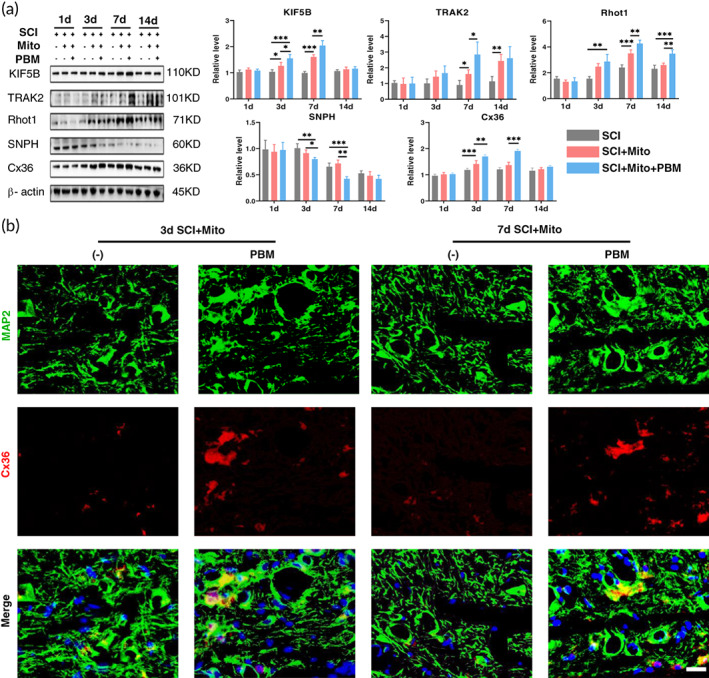
In the context of mitochondrial transplantation, PBM promoted the expression of Cx36, KIF5B, TRAK2, and Rhot1 and reduced the expression of SNPH. (a) Western blot analysis and quantification of KIF5B, TRAK2, Rhot1, SNPH, and Cx36 expression levels at 1, 3, 7, and 14 dpi in each group. *n* = 5. (b) Neurons stained with MAP2 (green) co‐localized with Cx36 (red) at 3, 7 dpi in SCI+Mito and SCI+Mito+PBM group. Nuclei were stained with DAPI (blue). Scale bar: 20 μm. *n* = 3.

The results indicated that Cx36 expression achieved a peak at 7 dpi in the SCI+Mito+PBM group but reached a transient peak at 3 dpi in the SCI+Mito group. There was no difference in Cx36 expression among the three groups at 14 dpi (Figure 3a *F*(6, 36) = 64.29, *p <* 0.001). The expression of TRAK2 peaked at 14 dpi, KIF5B and Rhot1 peaked at 7 dpi in the SCI+Mito group while they all peaked at 7 dpi in the PBM treatment group (Figure 3a, KIF5B: *F*(6, 36) = 40.03, *p* < 0.001; TRAK2: *F*(6, 36) = 13.5, *p <* 0.001; Rhot1: *F*(6, 36) = 16.99, *p <* 0.001). The protein level changes of these molecules are consistent with Cx36 which provides some support for Cx36‐mediated mitochondrial transplantation. In addition, PBM reduced the expression of SNPH from 3 to 7 dpi (*F*(6, 36) = 3.118, *p* = 0.01). Immunofluorescence results showed that PBM treatment could significantly increase the Cx36 level of neurons (MAP2, green) at 3 and 7 dpi (Figure 3b). This trend was consistent with the results of Western blotting of Cx36 (Figure 3a).

### 3.5 In the context of mitochondrial transplantation, PBM promoted the transfer of exogenous mitochondria into neurons

**FIGURE 4:**
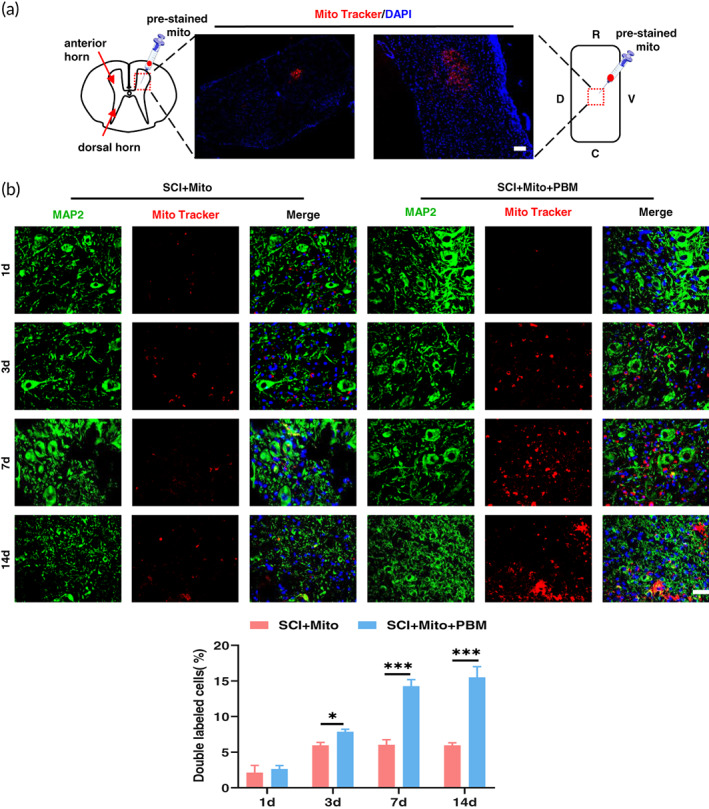
In the context of mitochondrial transplantation, PBM promoted the transfer of exogenous mitochondria into neurons. (a) The distribution of platelet‐derived mitochondria which was stained with Mito Tracker Red in the horizontal and sagittal planes of the injured segment. Nuclei were stained with DAPI (blue). R = Rostral; C = Caudal; D = Dorsal; V = Ventral. Scale bar: 100 μm. (b) Neurons stained with MAP2 (green) co‐localized with mitochondria (Mito Tracker Red) at 1, 3, 7, 14 dpi. Nuclei were stained with DAPI (blue). The ratio of mitochondria entering neurons was calculated. Scale bar: 20 μm. *n* = 4.

We further explored the effect of PBM on the transfer of exogenous mitochondria into neurons. First, we studied the distribution of mitochondria in the injured region. After SCI, Mito Tracker red pre‐stained functional mitochondria were injected into the spinal cords of rats, and the rats were killed 24 h later. Immunofluorescence showed that exogenous mitochondria were present in the injured parenchyma (Figure 4a). Figure 4b exhibited that PBM significantly increased the ratio of mitochondria that transferred to neurons. The proportion of double‐labeled neurons in the SCI+Mito+PBM group at 3 dpi was lower than that at 7 dpi (7.87 ± 0.35 vs 14.26 ± 0.92, *p* = 0.006) and there was no statistical difference between 7 and 14 dpi. This trend coincided with the dynamic trend of Cx36 after PBM intervention (Figure 3). However, there was no difference in the proportion of double‐labeled neurons in the SCI+Mito group between 3 and 7 dpi (5.96 ± 0.46 vs 6.03 ± 0.72, *p* = 0.77).

### 3.6 Combined therapy enhanced mitochondrial function after SCI at 14 dpi

**FIGURE 5:**
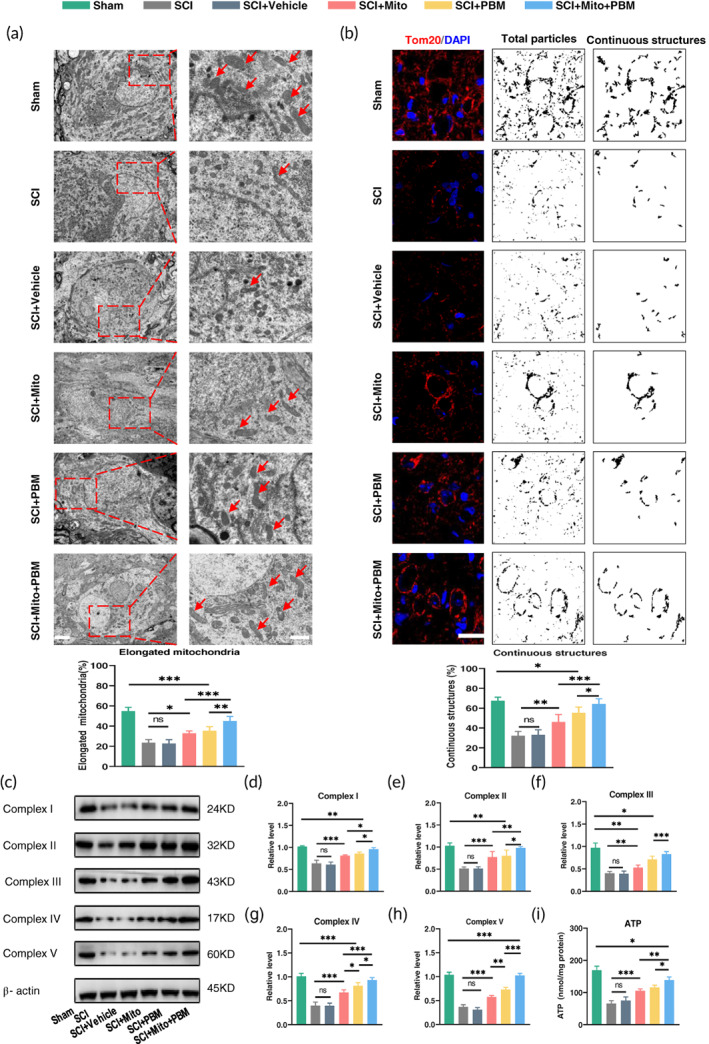
Combined therapy promoted mitochondrial activity. (a) Representative images of neuronal mitochondria in each group by TEM. Qualitative analysis of the proportion of elongated (>2 μm in size) mitochondria. Representative mitochondria are indicated by red arrows. Left panel scale bar: 2 μm. Right panel scale bar: 1 μm. *n* = 5. (b) Representative images of mitochondria stained with Tom20 antibody (red) at 14 dpi. Nuclei were stained with DAPI (blue). Images were separated, thresholded, filtered, and binarized with Image J. Continuous mitochondrial structures were calculated as the percentage of the area of large particles normalized to the total mitochondrial particles area. Scale bar: 10 μm. *n* = 5. (c–h) Western blot analysis and quantification of the expression levels of Complex I‐V in each group. *n* = 5. (i) Result of ATP levels in each group. *n* = 5.

Figure 4 clarified that the SCI+Mito+PBM group had a high ratio of functional mitochondria than the SCI+Mito group, and we then analyzed the ratio in all groups. TEM was used to assess the ratio of elongated mitochondria (size >2 μm), which were considered functional mitochondria to produce ATP.^48^, ^49^ There was no difference in the ratio of elongated mitochondria between the SCI+Mito group and the SCI+PBM group, while there was a significant difference between the SCI+Mito+PBM group and the other two treatment groups (Figure 5a, *F*(5, 24) = 58.45, *p <* 0.001). As shown in Figure 5b, Tom20 staining was used to calculate continuous mitochondrial structures (size >2 μm) as in the previous study.^50^ Continuous mitochondrial structures in the SCI+PBM group were no different from that in the SCI+Mito group but were lower than the SCI+Mito+PBM group (Figure 5b, *F*(5, 24) = 38.45, *p <* 0.001). The mitochondrial respiratory chain complex mediates oxidative phosphorylation and ultimately ATP production. Western blotting suggested that combined therapy could promote the recovery of mitochondrial complex activity (Figure 5c–h). The ATP content in the SCI+PBM group was not different from that in the SCI+Mito group (Figure 5i: 116.1 ± 6.4 vs. 105.2 ± 5.8, *p =* 0.45) but was still lower than that in the SCI+Mito+PBM group (116.1 ± 6.4 vs. 138.5 ± 9.7, *p =* 0.01).

### 3.7 Combined therapy alleviated oxidative stress at 14 dpi

**FIGURE 6:**
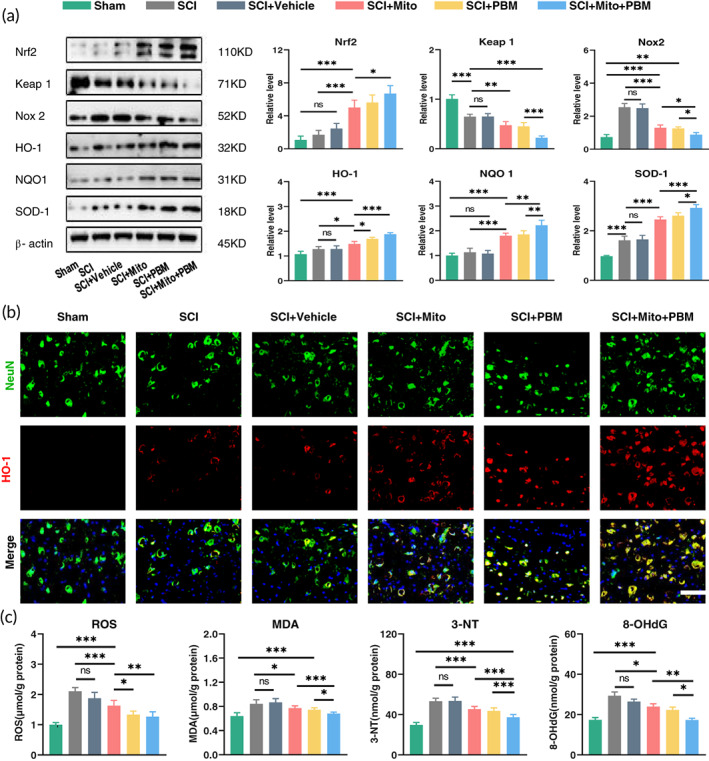
Combined therapy alleviated oxidative stress. (a) Western blot analysis and quantification of the expression levels of Nrf2, Keap 1, Nox2, HO‐1, NQO1, and SOD‐1 in each group. *n* = 5. (b) Representative images of NeuN (green) and HO‐1 (red) for each group. Nuclei were stained with DAPI (blue). Scale bar: 50 μm. (c) Results of 8‐OHdG, MDA, 3‐NT, and ROS in each group. *n* = 5.

Based on the close relationship between mitochondrial function and oxidative stress level, we further observed the level of oxidative stress. Nuclear erythroid 2‐related factor 2 (Nrf2) is an important transcription factor for antioxidant gene expression. The previous study has shown that Nrf2‐related pathways are the most significantly changed pathways after mitochondrial transplantation.^51^ Under normal physiological conditions, Nrf2 interacts with the binding protein Kelch‐like ECH‐associated protein 1 (Keap1) in the cytoplasm and rapidly degrades it through the ubiquitin‐proteasome pathway.^52^ During oxidative stress, Nrf2 activates downstream antioxidant enzymes such as heme oxygenase‐1 (HO‐1), superoxide dismutase‐1 (SOD‐1), and NAD(P)H quinone dehydrogenase 1 (NQO‐1) and inhabit NADPH oxidase 2 (NOX2) expression to maintain intracellular redox homeostasis.^52^, ^53^ Compared with the SCI group, combined therapy significantly reduced the level of Keap1 (Figure 6a: *F*(5, 24) = 80.13, *p <* 0.001) and increased the expression of Nrf2 (*F*(5, 24) = 46.62, *p <* 0.001). The high expression of Nrf2 upregulated the expression of HO‐1 (*F*(5, 24) = 46.05, *p <* 0.001), SOD‐1 (*F*(5, 24) = 165.4, *p <* 0.001), and NQO‐1 (*F*(5, 24) = 61.08, *p <* 0.001), while downregulated NOX2 expression (*F*(5, 24) = 96.93, *p <* 0.001). As shown in Figure 6b, the SCI group had a low expression of HO‐1 of neurons and these changes were reversed after treatment, especially in the combined therapy group. Analysis of ROS content showed that the level of ROS increased significantly after SCI but decreased significantly after treatment (Figure 6c: *F*(5, 24) = 40.86, *p <* 0.001). The levels of the lipid oxidative stress marker MDA, DNA oxidative stress marker 8‐OHdG, and protein oxidative stress marker 3‐NT increased significantly after injury, but these oxidative stress indexes decreased significantly after treatment (Figure 6c: 8‐OHdG: *F*(5, 24) = 62.89, *p <* 0.001; MDA: *F*(5, 24) = 53.03, *p <* 0.001; 3‐NT: *F*(5, 24) = 135.9, *p <* 0.001).

### 3.8 Inhibition of Cx36 reduced PBM‐promoted transfer of mitochondria to DRGs in vitro

**FIGURE 7:**
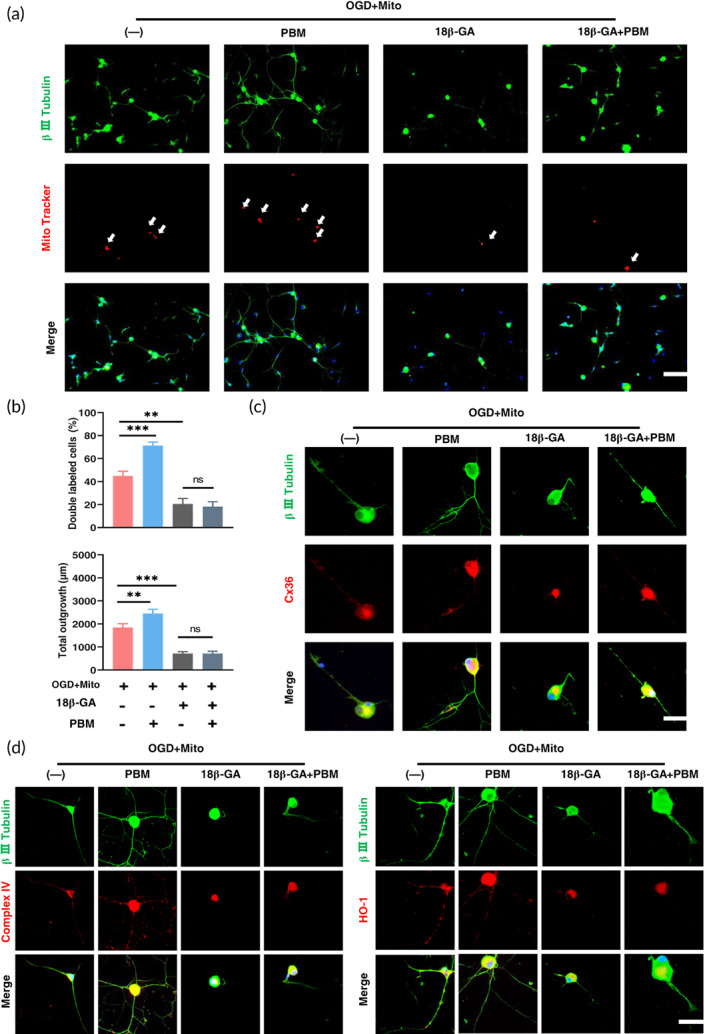
Inhibition of Cx36 reduced PBM‐promoted transfer of mitochondria in vitro. (a) DRG was treated with OGD for 4 h, and then treated with PBM or 18β‐GA (Cx36 inhibitor), finally, mitochondria (Mito Tracker Red pre‐stained) were added to co‐culture for 24 h. (b) The ratio of mitochondria entering DRG (β III Tubulin, green) and the length of axons were calculated by Image J. Scale bar: 20 μm. *n* = 5. (c) DRG staining of β III Tubulin (green) co‐localized with Cx36 (red). Scale bar: 50 μm. *n* = 3. (d) DRG staining of β III Tubulin (green) co‐localized with Complex IV (red), HO‐1 (red). Nuclei were stained with DAPI (blue). Scale bar: 50 μm; *n* = 3.

Finally, we further validate the effect of PBM on mitochondrial transfer in vitro. As shown in Figure 7a, b, the ratio of mitochondria that entered DRG neurons was lower (20.5 ± 4.79 vs 44.75 ± 4.27, *p* = 0.001) and the total axon length was shorter (715.6 ± 81.80 vs. 1838.2 ± 170.6, *p <* 0.001) in the group treated with Cx36 inhibitor (18β‐GA) than in the OGD+Mito group, indicating that connexin mediated the transfer of mitochondria into neurons. After PBM treatment, the ratio was higher (74.25 ± 4.34 vs. 44.75 ± 4.27, *p <* 0.001) and the total axonal length was longer (2447.1 ± 186.2 vs 1838.2 ± 170.6, *p <* 0.001) in OGD+Mito+PBM group compared with OGD+Mito group. In addition, the ratio was not higher and the axonal length was not longer in the group successively treated with inhibitors and PBM compared with the 18β‐GA only group (ratio: 20.5 ± 4.796 vs.18.10 ± 9.09, *p* = 0.91; axonal length: 715.6 ± 81.80 vs. 756.7 ± 65.12, *p* = 0.82). This trend was in line with the expression trend of Cx36 in Figure 7c. We further verified this conclusion in mitochondrial activity and its downstream antioxidant enzymes. After adding inhibitors, the expression of mitochondrial respiratory chain complex IV decreased while single PBM treatment increased the expression relative to the OGD+Mito group. Under the condition that PBM and inhibitors were both applied, the expression was not increased relative to the single inhibitor group (Figure 7d). The expression of HO‐1 showed the same trend as complex IV which further validated our results (Figure 7d).

## 4 DISCUSSIONS

SCI is a clinically challenging disorder, mostly caused by violent factors such as trauma and traffic accidents, resulting in the loss of motor function and even self‐care ability for most patients.^54^ The pathophysiology of SCI can be divided into two stages: primary injury and secondary injury. The primary injury includes direct destruction of spinal cord tissue by harmful stimuli such as hemorrhagic edema or compression. During this process, rupture of axonal membranes and elevation of the intracellular calcium ion concentration could lead to cell dysfunction, which could in turn induce mitochondrial depolarization in neurons.^55^ The secondary injury involves chronic inflammation, excitotoxicity, harmful oxidative reactions, and other pathological processes that ultimately lead to mitochondrial damage in neurons.^7^

Considering the key role of mitochondrial dysfunction in SCI, therapeutic strategies that target mitochondria are receiving increasing attention. Drug therapies that target a molecule or pathway related to mitochondria are not only limited but also difficult to reach the injury site due to the blood–spinal‐cord barrier. Therefore, direct supplementation of healthy mitochondria in the injury site might be helpful for SCI. However, this strategy does not meet the need for efficient transfer of mitochondria to neurons. Long‐term observations show that mitochondrial transplantation alone was not effective enough.^34^

In the past several decades, many researchers have tried to improve the efficiency of mitochondrial transfer to cells. The complex formed by the cell‐penetrating peptide pep‐1 and mitochondria could enhance the efficiency of cellular uptake of mitochondria in Parkinson's disease. In vivo, after intracerebral injection, the ratio of cells containing the labeled peptide was higher than that of unlabeled cells, and the neuroprotective effect of the labeled group was better.^56^ However, cell‐penetrating peptides have not been used in the clinic. Recent studies have shown that a stem cell delivery vector promoted the expression of connexin and could improve the efficiency of mitochondrial transfer to damaged cells.^57^ But there are still many ethical problems related to the clinical use of stem cells. The optical fiber developed by our research group has been successfully applied in SCI patients and it was proven to be safe and efficinetly.^58^ Therefore, we began to explore whether PBM could expand the therapeutic effect of single mitochondrial transplantation and its mechanism.

First, we clarified that the combined therapy had an obvious effect on motor function recovery, tissue repair (Figure 1), and the reduction in neuronal apoptosis (Figure 2). Second, we found that after PBM intervention, the trend of exogenous mitochondria entering neurons (Figure 4) was consistent with the expression trend of Cx36 (Figure 3). Notably, Tom 20 (Figure 5) and TEM (Figure 5) analysis showed that there was no difference in the ratio of functional mitochondria in the two single treatment groups. Considering that each harmful pathological response after SCI will hinder the recovery of mitochondrial function by each treatment method, which is, in the absence of other effects of these two treatment methods, simple superposition therapy will not significantly increase the ratio of functional mitochondria. Moreover, the ratio of functional mitochondria in the combination therapy group increased compared with that in the single treatment group. We assumed that the increased portion of mitochondria came from exogenous, which meant the ratio of exogenous mitochondria entering neurons increased after the PBM intervention. Further research showed that the combination treatment performed best in terms of ATP production (Figure 5) and antioxidant capacity by upregulating the Nrf2 pathway (Figure 6).

Functional mitochondrial increase after mitochondrial transplantation and Nrf2 expression are two mutually regulated processes. On one hand, mitochondrial transplantation reduces oxidative stress by upregulating Nrf2.^59^ Mitochondria are the main organelles of antioxidant stress, and the Nrf2 pathway is the main part of the body's antioxidant defense system that can enhance cell resistance to oxidative stress. The increase in the number of functional mitochondria activated this pathway to play an antioxidant role.^59^, ^60^, ^61^, ^62^, ^63^ More specifically, Peroxisome proliferators‐activated receptor γ coactivator alpha (PGC‐1α), an important gene transcription coactivator, can activate Nrf2 gene transcription, coordinate mitochondrial respiratory chain function, and regulate the level of oxidative stress. On the other hand, the increased expression of Nrf2 is also one of the mechanisms to protect mitochondria from apoptosis after transplantation.^64^ Nrf2 controls the level of active oxygen free radicals by up‐regulating the expression of antioxidant genes, inhibits the decline of mitochondrial membrane potential, protects mitochondrial functions such as ATP synthesis, and maintains redox homeostasis after transplantation.^59^

Third, we designed in vitro experiments to validate our assumption. After DRGs were treated with a Cx36 inhibitor, the ratio of mitochondria transferred to DRGs decreased, and the expression of mitochondrial respiratory chain complex IV and antioxidant enzymes HO‐1 was reduced while single PBM treatment increased it. Remarkably, there was no difference between the inhibitor group and the group cotreated with inhibitor and PBM in terms of the projects mentioned above. This meant that PBM could not promote the transfer progress after Cx36 was blocked. Therefore, Cx36 mediates the transfer of mitochondria into DRGs and PBM promotes this process (Figure 7).

**FIGURE 8:**
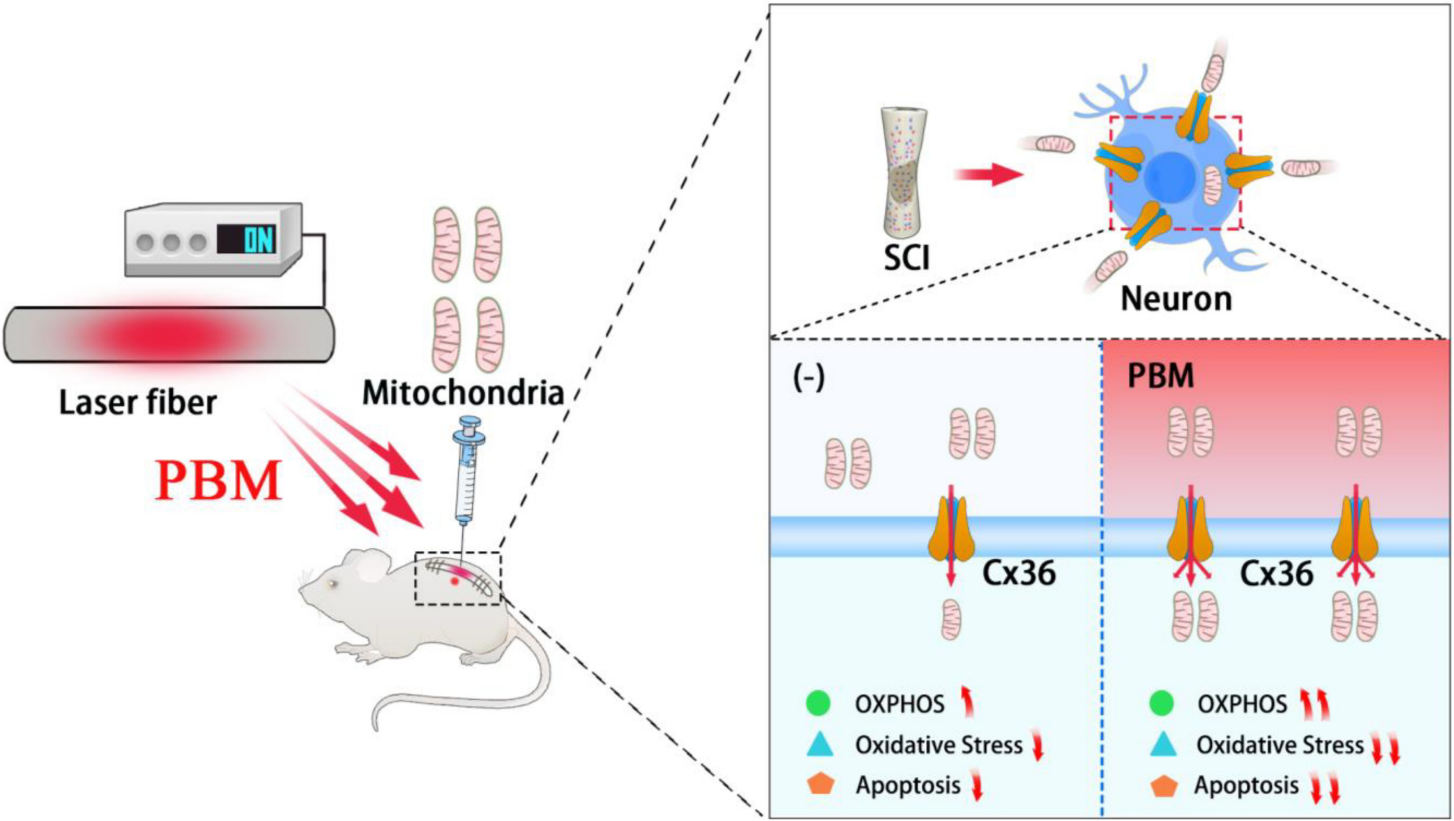
In vivo‐implanted laser fiber mediates PBM promoting the transfer of mitochondria to neurons by upregulating Cx36. Combined therapy increased ATP content, alleviated oxidative stress, and reduced neuronal apoptosis, thereby promoting tissue repair and motor function recovery after SCI. The picture was exported by FigDraw.

According to the results of the in vivo and in vitro experiments, we speculated that PBM promoted mitochondrial transfer by upregulating the expression of Cx36, compensating for the inability of simple mitochondrial transplantation because of the transient expression of Cx36 (Figure 8).

Of course, this study has some limitations. First, using gene knockout mice to verify mechanisms can increase persuasiveness. Second, mitochondria enter neurons through multiple pathways, and the influence of PBM on other pathways is not clear. Third, this article is only verified in rat models, an in‐depth study of the effects and mechanisms in large animal models is the premise of the future clinical application of this technology, which is urgent for further investigation.

In summary, our work indicated that PBM promoted the transfer of mitochondria into neurons and augmented the effects of mitochondrial transplantation (Figure 8). This combined treatment also provides new insight for clinical research.

## AUTHOR CONTRIBUTIONS

**Zhijie Zhu:** Conceptualization (equal); data curation (lead); formal analysis (equal); investigation (equal); methodology (equal); writing – original draft (equal); writing – review and editing (equal). **Xin Li:** Conceptualization (equal); data curation (equal); investigation (lead); methodology (equal). **Xuankang Wang:** Data curation (equal); investigation (equal); methodology (equal). **Xiaoshuang Zuo:** Conceptualization (supporting); data curation (equal); methodology (equal). **Yangguang Ma:** Conceptualization (supporting); investigation (equal). **Xue Gao:** Conceptualization (supporting); investigation (equal). **Zhuowen Liang:** Conceptualization (supporting); investigation (supporting). **Zhihao Zhang:** Conceptualization (supporting); investigation (supporting). **Zhiwen Song:** Conceptualization (supporting); investigation (supporting). **Tan Ding:** Conceptualization (supporting); investigation (supporting). **Cheng Ju:** Conceptualization (supporting); investigation (supporting). **Penghui Li:** Conceptualization (supporting); investigation (supporting). **Kun Li:** Conceptualization (supporting); funding acquisition (supporting). **Jiawei Zhang:** Conceptualization (supporting); investigation (supporting). **Huilin Quan:** Conceptualization (equal); investigation (equal). **Zhe Wang:** Conceptualization (equal); funding acquisition (equal); investigation (equal); methodology (equal); project administration (equal); resources (equal); supervision (equal); validation (equal); writing – original draft (lead). **Xueyu Hu:** Conceptualization (lead); data curation (equal); formal analysis (equal); funding acquisition (lead); investigation (equal); methodology (equal); project administration (lead); supervision (lead); validation (equal); visualization (equal); writing – original draft (lead); writing – review and editing (lead).

## CONFLICT OF INTEREST

The authors declare no conflict of interest.

### PEER REVIEW

The peer review history for this article is available at https://publons.com/publon/10.1002/btm2.10473.

## Supporting information


**DATA S1.** Supporting Information

## Data Availability

The data used to support the findings of this study are available from the corresponding author upon request.
