# Supplementary material for: Photobiomodulation augments the effects of mitochondrial transplantation in the treatment of spinal cord injury in rats by facilitating mitochondrial transfer to neurons via Connexin 36
Source: Bioeng Transl Med. 2022 Dec 21;8(3):e10473. doi: 10.1002/btm2.10473 (PMC10189468; doi:10.1002/btm2.10473)
Supplement: Supplementary file 1 — DATA S1. Supporting Information [file BTM2-8-e10473-s001.docx]

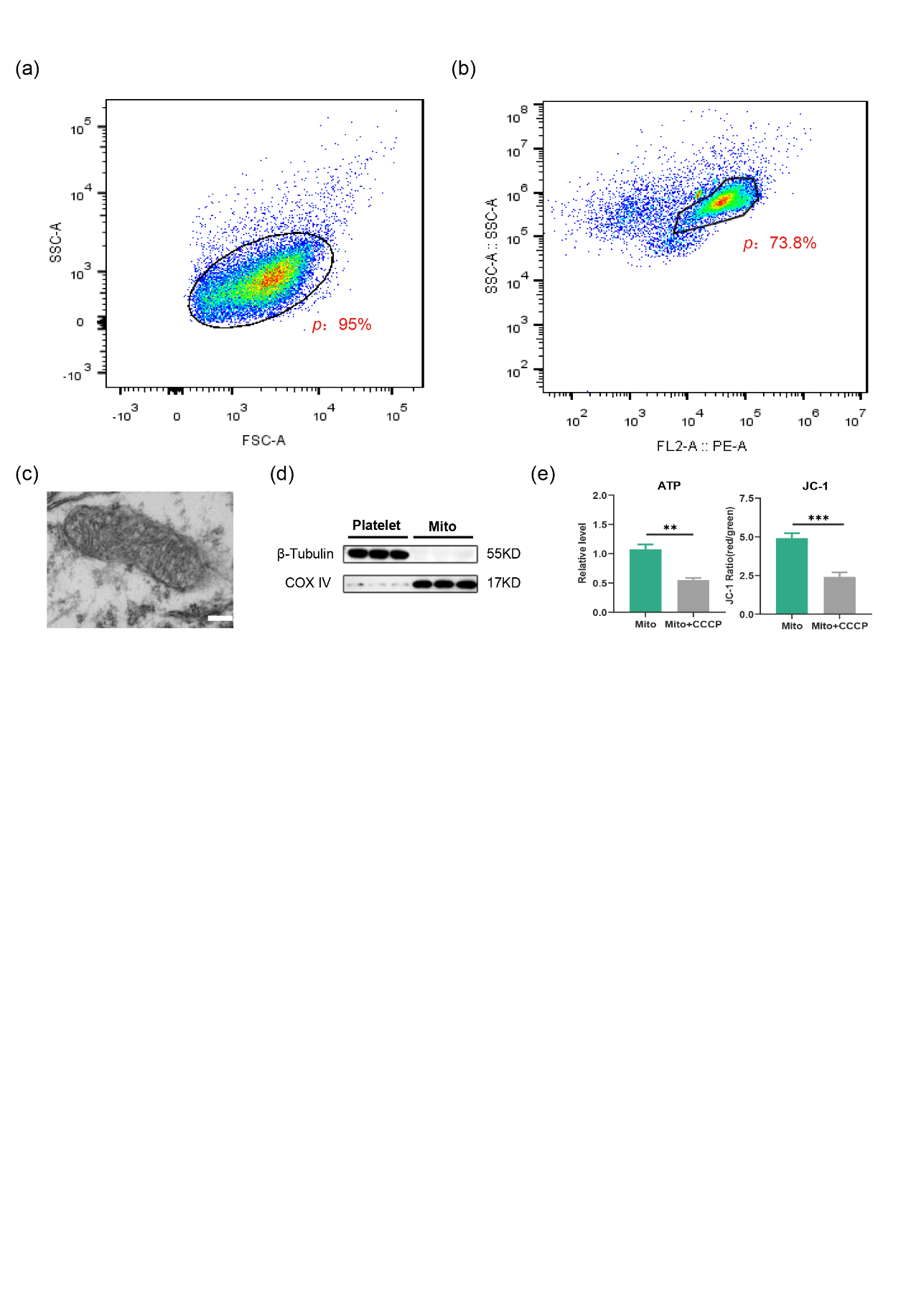


**Supplementary Figure 1: Identification of the properties of platelet-derived mitochondria.**

(a): Detection of platelet purity by flow cytometry. n=3. (b): Count the number of mitochondria by flow cytometry. n=3. (c): TEM showed platelet-derived mitochondria. Scale bar: 0.2µm. (d): Qualitative of platelet proteins and mitochondrial proteins by Western blot (n=3). (e): Purified mitochondrial ATP production and JC-1 assay, CCCP is an inhibitor as control. n=3.


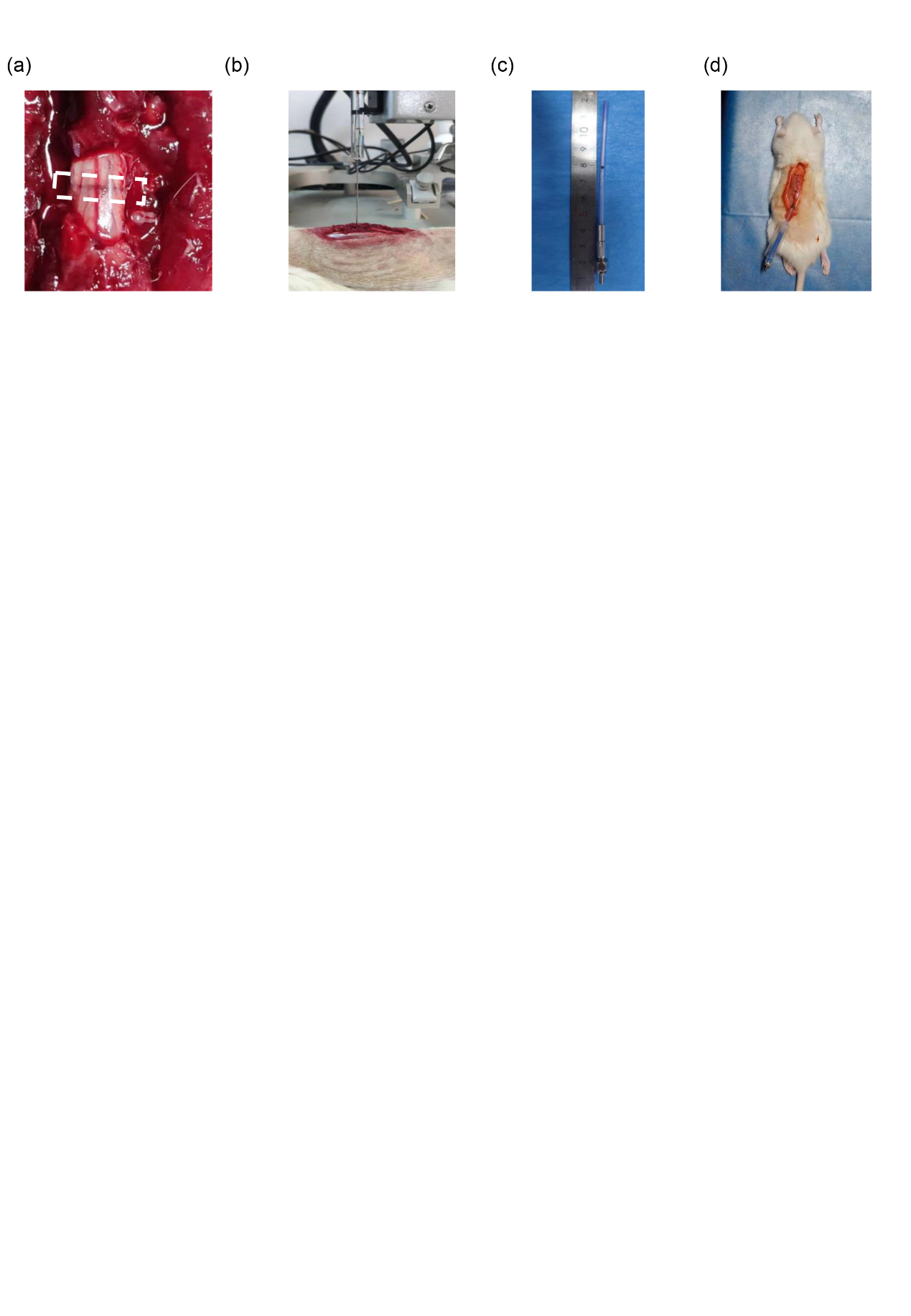


**Supplementary Figure 2: Animal models, mitochondrial injection and photobiomodulation therapy**

(A): Spinal cord injury crush model, the white rectangle area is the injury area. (B): Stereotactic injection of mitochondria. (C): Laser fiber outlook. (D): The fiber was embedded in the spinal cord surface.


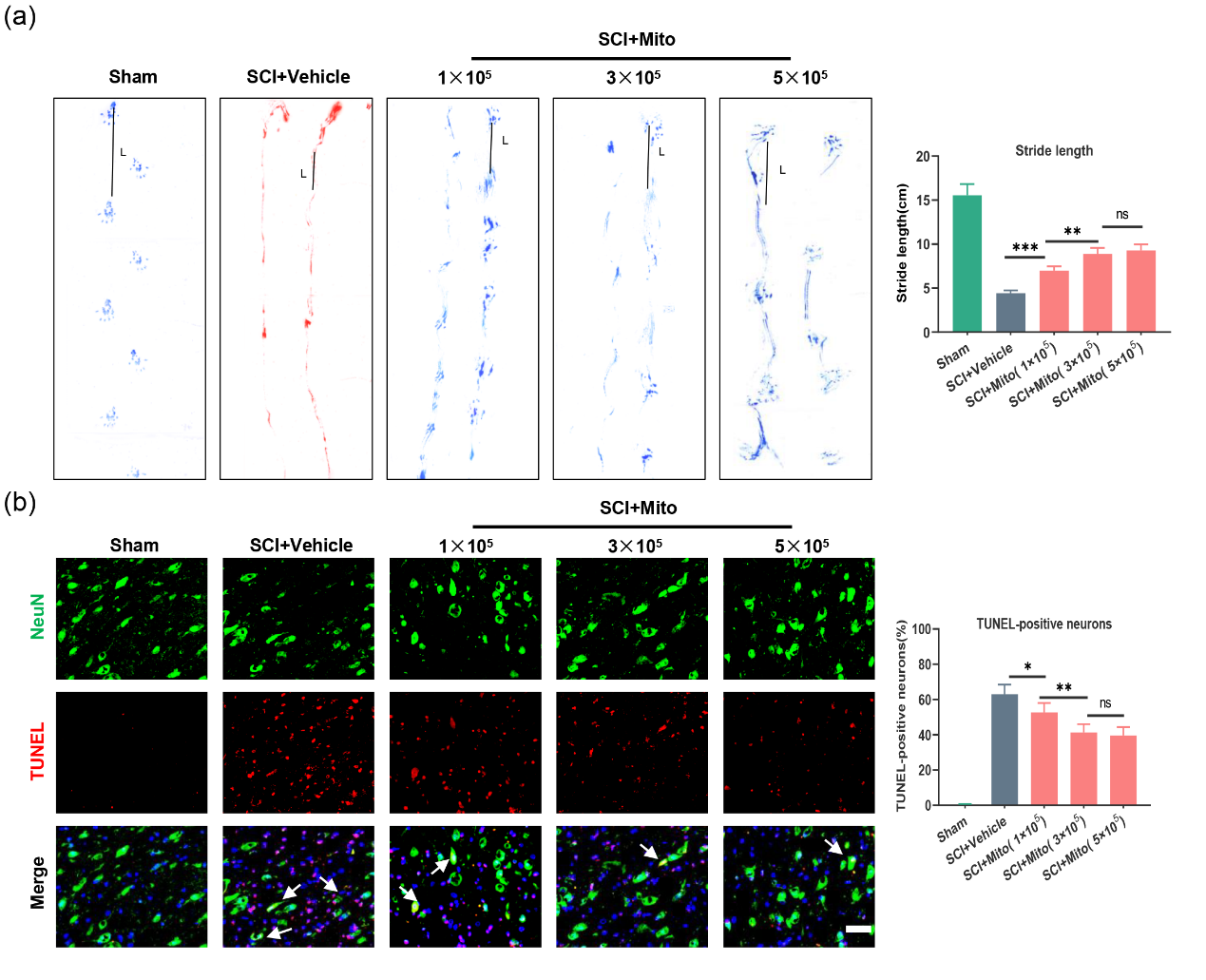


**Supplemental figure 3. Effects of different doses of mitochondrial transplantation on motor function and neuron apoptosis at 14 dpi.**

(a): Gait analysis and representative gaits of each group. L= stride length, n=4. (b): TUNEL (red) and NeuN (green) are co-labeled. Scale bar: 50µm. Arrows point to representative cells. n=4.

**Supplementary Table 1:Laser irradiation parameters.**

| **Parameter** | **Value** | |
| --- | --- | --- |
|  | in vivo | in vitro |
| Center wavelength (nm) | 810 | 810 |
| Spectral bandwidth (nm) | <3 | <3 |
| Operating mode | Continuous wave | Continuous wave |
| Frequency (Hz) | 50 kHz | 50 kHz |
| Beam spot size at target (cm²) | 0.3 | 4.5 |
| Irradiance at garget (mW/cm²) | 500 | 1.33 |
| Exposure duration (s) | 3600 | 600 |
| Radiant exposure (J/cm²) | 1800 | 0.64 |
| Radiant energy (J) | 540 | 2.88 |
| Number of points irradiated | 1/day | 2/day |
| Area irradiated (cm²) | 0.3 | 4.5 |

| **Supplementary Table 2. Antibodies used for western blot assays** | | | |
| --- | --- | --- | --- |
| Antibody name | Company | Catalog number | Concentration |
| Anti-NDUFB8（Complex I） | Abcam | ab192878 | 1:2000 |
| Anti-SDHB（Complex Ⅱ） | Abcam | ab175225 | 1:2000 |
| Anti-UQCRC2（Complex Ⅲ） | absin | abs116449 | 1:3000 |
| COX IV Antibody （Complex IV） | CST | 4844S | 1:3000 |
| ATP5F1A Antibody （Complex Ⅴ） | CUSABIO | PA002344HA01HU | 1:2000 |
| NRF2, NFE2L2 Polyclonal Antibody | Proteintech | 16396-1-AP | 1:1000 |
| KEAP1 Polyclonal Antibody | Proteintech | 10503-2-AP | 1:2000 |
| Anti-NOX2/gp91phox | Abcam | ab129068 | 1:2000 |
| Anti-Heme Oxygenase 1 | Abcam | ab189491 | 1:2000 |
| Anti-NQO1 | Abcam | ab80588 | 1:2000 |
| SOD1 Polyclonal Antibody | Proteintech | 10269-1-AP | 1:2000 |
| BAX Polyclonal Antibody | Proteintech | 50599-2-Ig | 1:1000 |
| Bcl2 Polyclonal Antibody | Proteintech | 26593-1-AP | 1:2000 |
| Caspase-3 Antibody | CST | 9662S | 1:2000 |
| Caspase 3/P17/P19 Antibody | Proteintech | 19677-1-AP | 1:1000 |
| Myelin Basic Protein mAb | CST | 78896S | 1:2000 |
| Anti-GAP43 | Abcam | ab75810 | 1:2000 |
| TRAK2 Polyclonal Antibody | Proteintech | 13770-1-AP | 1:1000 |
| Rhot1 Antibody | NOVUS | NBP1-89011 | 1:2000 |
| KIF5B Polyclonal Antibody | Proteintech | 21632-1-AP | 1:2000 |
| Syntaphilin Polyclonal Antibody | Proteintech | 13646-1-AP | 1:2000 |
| Connexin 36 Polyclonal Antibody | Thermo Fisher | 51-6200 | 1:500 |
| Beta Actin Monoclonal Antibody | Proteintech | 66009-1-Ig | 1:3000 |
| Beta Tubulin Monoclonal Antibody | Proteintech | 10068-1-AP | 1:3000 |
